# Prevalence, Awareness, and Control of Hypertensive Disorders amongst Pregnant Women Seeking Healthcare in Ghana

**DOI:** 10.1155/2023/4194443

**Published:** 2023-09-12

**Authors:** Pauline Boachie-Ansah, Berko Panyin Anto, Afia Frimpomaa Asare Marfo, Edward Tieru Dassah, Constance Caroline Cobbold, Morrison Asiamah

**Affiliations:** ^1^Department of Pharmacy Practice, Faculty of Pharmacy and Pharmaceutical Sciences, Kwame Nkrumah University of Science and Technology, Kumasi, Ghana; ^2^Department of Population and Family Reproductive Health, School of Public Health, Kwame Nkrumah University of Science and Technology, Kumasi, Ghana; ^3^Department of Obstetrics and Gynaecology, Komfo Anokye Teaching Hospital, Kumasi, Ghana; ^4^Department of Pharmacotherapeutics and Pharmacy Practice, School of Pharmacy and Pharmaceutical Sciences, University of Cape Coast, Ghana; ^5^Department of Electron Microscopy and Histopathology, Noguchi Memorial Institute for Medical Research, University of Ghana, Legon, Accra, Ghana

## Abstract

Hypertensive disorders in pregnancy (HDPs) are no longer seen as “transitory diseases cured by delivery.” It accounts for up to 50% of maternal deaths. Information concerning HDPs is less in developing countries like Ghana. This study was conducted to find out the prevalence, awareness, risk factors, control, and the birth outcomes of HDPs. This was a retrospective cohort study conducted among pregnant women seeking care in selected health facilities in the Ashanti Region. Data on demographics, HDPs, and its associated birth outcomes were collected. Logistic regression models were used to examine the association of the independent variables with HDPs. The burden of HDPs was 37.2% among the 500 mothers enrolled with chronic hypertension superimposed with preeclampsia accounting for 17.6%, chronic hypertension, 10.2%, and preeclampsia 6.8% whilst gestational hypertension was 2.6%. It was observed that 44% (220) of the mothers had excellent knowledge on HDPs. Oral nifedipine and methyldopa were frequently used for HDP management, and it resulted in a significant reduction in HDP burden from 37.2% to 26.6%. Factors that influenced the increased risk of HDPs were grand multigravida (AOR = 4.53; CI = 1.42–14.42), family history of hypertension (AOR = 3.61; CI = 1.89–6.90), and the consumption of herbal preparations (AOR = 2.92; CI = 1.15–7.41) and alcohol (AOR = 4.10; CI = 1.34-12.62) during pregnancy. HDPs increased the risk of preterm delivery (AOR = 2.66; CI = 1.29–5.89), stillbirth (AOR = 12.47; CI = 2.72–57.24), and undergoing caesarean section (AOR = 1.70; CI = 1.10–2.61) amongst mothers during delivery. The burden of HDPs is high amongst pregnant mothers seeking care in selected facilities. There is the need for intensified campaign on HDPs in the Ashanti Region of Ghana.

## 1. Background

Hypertensive disorders in pregnancy (HDPs) are the leading cause of maternal mortality [1]. It contributes to 14% and 16% maternal mortality worldwide and in Sub-Saharan Africa (SSA), respectively [2]. Chronic hypertension, preeclampsia-eclampsia, gestational hypertension, and chronic hypertension-superimposed preeclampsia are the four categories of hypertensive disorders in pregnancy [3]. In Ghana, it is estimated that the maternal mortality rate is 319 deaths per 100,000 live births [4]. This is about 4.3 times higher than the UN recommended rate. One of commonest cause of direct maternal death identified was severe preeclampsia/eclampsia which accounted for 23% of all direct maternal deaths and 16% of all maternal deaths in 2011 [5].

Factors such as educational level, age, higher parity, occupation, family of HDPs, gravida, complications of pregnancy, and others such as healthcare systems and social, cultural, and economic factors are seen to be associated with HDPs [6]. Studies have shown that a woman's knowledge about her condition can influence early health seeking behaviour and compliance to treatment which would result in early diagnosis and prevent pregnancy complication including death [7]. Lack of knowledge on HDPs could lead to misconceptions which may include interpretation of warning signs as normal occurrence during pregnancy or as “spiritual attacks” [8]. Although maternal serum AQP9 concentrations have been found to be associated with early-onset preeclampsia [9], the cause of HDPs remains unclear, and treatment remains symptomatic with the primary objective being the safety of the mother. Over the years, HDPs have being controlled with regular blood pressure monitoring, use of approved pharmacotherapeutics, and other nonpharmacological preventive measures.

Target 3.1 of the Sustainable Development Goals (SDG) as set by the United Nations is aimed at reducing the maternal mortality ratio to 70 per 100,000 live births by 2030 [10].

However, the burden of hypertension has been increasing over the past few decades in SSA, and a large percentage of the population with hypertension remains untreated, ineffectively treated, or even undiagnosed [11]. Furthermore, this study is important because much research has studied the prevalence, awareness, and control of hypertension among the general population; however, there is a need for studies focused on pregnant women as the effect of HDPs include risk of harm not only to the mother but also to the unborn child. HDPs are no longer seen as “transitory diseases cured by delivery,” but as windows into the woman's future health that needs integrated care, coordinating treatment to identify risk factors and correct them [12]. This study is aimed at identifying the prevalence, risks awareness, control, and the birth outcomes of HDPs among pregnant women in some selected health facilities in the Ashanti Region of Ghana.

## 2. Methods

### 2.1. Study Design and Site

This was a retrospective cohort study, which employed a well-structured, pretested, close-response questionnaire to obtain data from pregnant women who were seeking care at four hospitals in the Ashanti Region. The Ashanti Region is the second most populous region in Ghana (GSS 2019) with over 100 hospitals, both private and public hospitals. The study sites included the Ejisu Government Hospital (Ejisu Municipal), Kwame Nkrumah University of Science and Technology (KNUST) Hospital (Oforikrom Municipal), Kumasi South Hospital (Asokwa Municipal), and the Komfo Anokye Teaching Hospital (KATH) (Kumasi Metropolis, Bantama sub-metro district). These facilities were chosen because of their location in the region and the large coverage of patients they cater for.

### 2.2. Sample and Sampling

This study included pregnant women above the ages of 18 years who had been admitted for delivery and those who had delivered at the hospital during the period of the study. Pregnant women who were on admission for other reasons besides delivery were excluded.

At a significance level of 5%, the Yamane method was used to determine the sample size [13]. The estimated number of live births in the Ashanti Region in 2010 was 122878 [14]. If the number of live births equals the number of pregnant women for the year, a sample size of 440 pregnant women would be sufficient. Considering nonresponse rate, a minimum of about 500 pregnant women were recruited. From this figure, 105 (21%), 125 (25%), 120 (24%), and 150 (30%) mothers were recruited from the Ejisu District Hospital, KNUST Hospital, Kumasi South Hospital, and KATH, respectively. Employing quota, sample size for each facility was determined using the documented average monthly delivery at each hospital.

Consented participants were screened for eligibility. Participants who were eligible for this study were numbered and randomly sampled using an online number generator. Participants whose numbers were generated were included in the study.

### 2.3. Development and Validation of Questionnaire

The questionnaire was developed after reviewing several literatures to ensure that the items capture a meaningful construct to have causality to the outcome of interest. The questionnaire was piloted within a selected hospital. To assess the reliability of the construct of composite variables such as the level of knowledge, a Cronbach alpha test was conducted. For the Cronbach alpha test for level of knowledge, the score was 0.73 which indicates that the composite variable is reliable. Information was collected on their sociodemographic characteristics, factors influencing their risk of hypertensive disorders in pregnancy, level of awareness, management of the disorders, and the birth outcomes.

The level of knowledge was assessed as an 18-item composite variable. For a correct response a code of 1 was assigned, and for a wrong response, a code of 0 was assigned. Therefore, a participant can score a maximum of 18 and a minimum of 0. Where a participant scores less than 5, and then, the participant has poor level of knowledge, scoring between 5 and 9 was considered satisfactory level of knowledge, and 10 to 14 was considered good level of knowledge. A participant is considered to have an excellent level of knowledge on HDPs if the participant scores 15 to 18.

Code of 1 was assigned to participants who had HDPs and 0 to participants who did not have evidence of HDPs. Thus, the dependent variable hypertensive disorder in pregnancy was measured as a binary outcome.

### 2.4. Data Collection

A research team comprising medical officers, pharmacists, pharmacy house officers, research scientists, and midwives was formed and oriented through series of meetings on the procedures and the objectives of the study. Each member had a specific role to play. The team was grouped into four smaller groups for each hospital. All consenting women who had delivered had their antenatal record booklets reviewed retrospectively from 16 week gestation to the point of delivery by a member of the research team and underwent a confidential face-to-face interview (where possible) in vernacular or English using a pretested structured questionnaire. The questionnaire was used to collect data on demographic, socioeconomic characteristics, patient-related factors, and knowledge on hypertension related disorders in pregnancy (HDPs). Antenatal record books were reviewed for the outcome and mode of pregnancy, blood pressure control during the gestational period, antenatal quality indices, and any diagnosis of HDPs. The mode of deliveries was recorded in patients' records by physicians or the midwives.

Women who had been admitted for delivery but had not delivered were recruited after delivery and then interviewed. The participants were allowed to withdraw from the study at any time if they wish to do so and reasons for withdrawal documented.

### 2.5. Data Analysis

Data was entered in Excel and cross-checked, cleaned, and exported to Stata version 14 for the analysis. There were no missing data due to the use of interviewer administered questionnaire and a check on women medical records. A univariate analysis employing the Pearson's Chi square test and simple logistic regression analysis was carried out to assess the factors associated with HDPs. Multiple logistic regression analysis was considered to assess the association between independent variables that proved to be statistically significant under the univariate analysis. The results obtained from the multivariable analysis were expressed as odds ratio with their respective 95% confidence intervals (CIs) and *p* values. Statistically significant was considered at a *p* < 0.05.

## 3. Results

### 3.1. Sociodemographic Characteristics of Respondents

A total of 500 pregnant women participated in this study. A fifth of the participants were aged 18 to 24 years, 174 (34.8%) aged between 25 and 29 years, and a quarter were aged 30 to 34 years. Respondents were more likely to be educated (420; 84.0%), married (378; 75.5%), Christian (394; 82.8%), managing their own private businesses (320; 64.8%), prefer to receive antenatal care (ANC) in a health facility located in an urban area (270; 54.0%), and have some knowledge on HDPs (429; 85.80%) (Table 1).

### 3.2. Medical Characteristics of Respondents

Women attending the health facilities were more likely to be multigravida (329; 66.06%), with no history of miscarriage (400; 80.16%) nor caesarean section (318; 63.60%), and do not have history of chronic condition (420; 85.80%), nor HDPs (337; 67.54%). Most of them, 342 (68.67%), had their first antenatal visit in their first trimester (Table 2).

### 3.3. Prevalence of Hypertensive Disorders in Pregnancy (HDPs)

**Figure 1:**
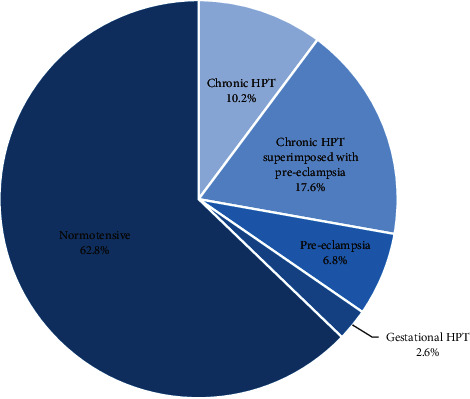
Proportion of various types of HDPs amongst study respondents.

The results indicated that, out of the 500 respondents, 314 (62.8%) had no hypertension throughout their pregnancy while 186 (37.2%) were with HDPs. Fifty-one (27.4%) were chronic hypertensive, chronic hypertension superimposed with preeclampsia was 88 (17.6%), preeclampsia was 34 (6.8%), and gestational hypertension was 13 (2.6%) (Figure 1).

### 3.4. Antihypertensives Prescribed and Blood Pressure Control among Women with HDPs

**Figure 2:**
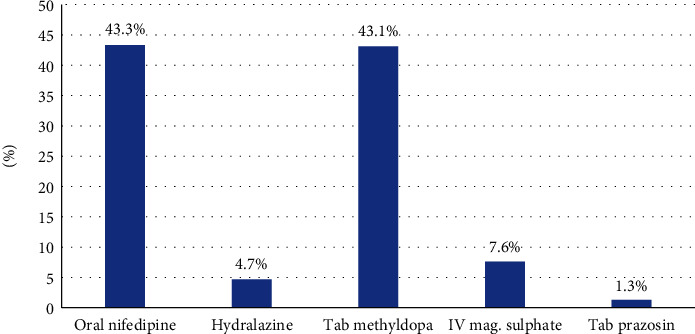
Medicines used to manage HDPs in the selected health facilities.

All women diagnosed of HDPs received at least one antihypertensive. Oral nifedipine (43.3%, *n* = 81) and oral methyldopa (43.1%, *n* = 80) were often prescribed, 7.6% (*n* = 14) of respondents received IV magnesium sulphate, 4.7% (*n* = 09) were on injection, and hydralazine and tab prazosin were 1.3%. The parenteral medications were prescribed during pregnancy for those who recorded high blood pressure or were at risk of developing eclampsia (Figure 2).

### 3.5. Effect of Intervention on the Blood Pressure Control of Respondents

The mean blood pressure of patients with HDPs was 137/93 mmHg at 16 week gestation. However, at the time of delivery, the mean blood pressure was 137/89 mmHg. The reduction in blood pressure due to medicine treatment was not statistically significant. However, there was a significant reduction in the proportion of mothers who had hypertensive disorders from 37.2% to 26.6% (Table 3).

### 3.6. Factors Influencing Hypertensive Disorders in Pregnancy

From the findings, mothers who had education at the junior high school level were 68% less likely to have HDPs compared to mothers who had no education (AOR = 0.27; CI = 0.10–0.68). Grand multigravida mothers were 4.5 times more likely to have HDPs compared to primigravid mothers (AOR = 4.53; CI = 1.42–14.42). Additionally, mothers with family history of hypertension were 4.3 times more likely to have HDPs (AOR = 3.61; CI = 1.89–6.90). Herbal consumption during pregnancy was associated with an increased risk of HDPs (AOR = 2.92; CI = 1.15–7.41), and also, mothers who consumed alcohol were more likely to have HDPs (AOR = 4.10; CI = 1.34-12.62) (Table 4).

### 3.7. Association between Hypertensive Disorders and Obstetric Outcomes

Tables 5–7 present the findings on the association between hypertensive disorders in pregnancy and various obstetric outcomes. Mothers with HDPs were 2.66 times more likely to have preterm birth compared to mothers who were not diagnosed of HDPs (AOR = 2.66; CI = 1.29–5.89). The health facility, alcohol consumption status, and family history of HDPs did not influence the risk of preterm delivery (Table 5).

Similarly, mothers who are diagnosed of HDPs have 70% increased chance of experiencing caesarean section mode of delivery compared to mothers who were not diagnosed of HDPs (AOR = 1.70; CI = 1.10–2.61). Mothers who consumed alcohol were 2.37 times more likely to experience caesarean section compared to mothers who did not consume alcohol (AOR = 4.21; CI = 2.37–7.49). Mothers who had family history of hypertension were 1.61 times more likely to undergo caesarean section during delivery (AOR = 1.61; CI = 1.06–2.46) (Table 6).

In addition to the birth outcomes of the study, it was realized that mothers who were diagnosed of HDPs were 12.47 times more likely to have stillbirth during delivery compared to mothers who were not diagnosed of HDPs (AOR = 12.47; CI = 2.72–57.24). Other predictors such as maternal age, anxiety over pregnancy, history of chronic medical condition, and consumption of alcohol did not influence the risk of stillbirth amongst mothers (Table 7).

## 4. Discussion

Hypertensive disorders in pregnancy (HDPs) have been established to be a leading cause of maternal deaths, accounting for about 26.4% to 50% of all maternal deaths in the country [15]. In a study conducted in a teaching hospital in Ghana in 2017, the burden of HDPs was found 21.4% which is about two times the global burden [16]. With a national maternal mortality of 776 in 2020 [17], it was estimated that up to 388 maternal deaths in Ghana were caused by hypertensive disorders.

The burden of HDPs in this study was 37.2%, and 62.8% of the participants had no HDPs, which is comparable to a study by Awuah et al. [18], in which the burden of HDPs was 39.25% and the proportion of women without HDPs was 60.7%. Although the sample size was less compared to this study, the prevalence of gestational hypertension and preeclampsia were high compared to chronic hypertension in pregnancy. Awuah recorded 52% and 33% for preeclampsia and gestational hypertension, respectively, against 6.8% and 2.6% in this study. Another study which was conducted in a tertiary hospital in the Ashanti Region also recorded a prevalence of 32.4% and 48.8% for gestational hypertension and preeclampsia, respectively [19]. A systematic review of HDPs in Ethiopia pooled a national prevalence of 6.07 with subgroup analysis by region showing a higher prevalence of HDPs [20]. This confirms the high prevalence of HDPs in the region, and the needs for better strategies for prevention to improve pregnancy outcomes are required in the maternity care centers.

The disparity of the disease burden among the various studies could be attributed to a lack of consensus on the diagnostic criteria of the various hypertensive disorders. This is because the health facilities in this study all use treatment protocols similar to the national standard treatment guidelines [21] for management of HDPs. However, the blood pressure level to start treatment depends on the facility, prescriber on duty, and other patient-related factors [16]. It would be appropriate to address these discrepancies so that accurate prevalence can be recorded and tackled well.

Despite the above explanation, Ghana has often recorded a high burden of HDPs, and this should be of national health concern because it has been a major cause of maternal and prenatal morbidity and mortality.

Knowledge of an individual on a particular health condition influences the health seeking behaviour of individual [22]. Thus, knowledge on HDPs has been assessed as efforts at preventing HDP burden. It is evident from this study that almost half (44%) of the women have excellent knowledge of HDPs. This is very encouraging. Having more knowledge would mean that the mother would be informed on the causes, risk factors, signs, symptoms, and possibly treatment options for HDPs. Considering the negative effect of these disorders on both maternal and neonatal outcomes coupled with the associated economic cost of management, an increase in knowledge would go a long way to decrease the burden of the disorders, improve treatment compliance, and overall reduce maternal and neonatal morbidities and mortalities associated with the disorders. It is expected that as the knowledge of an individual increases, the risk of contracting that disease reduces.

Oral nifedipine and oral methyldopa, at different doses, were the drugs often prescribed. This finding is similar to the findings of Kumar et al. where it was observed that both nifedipine and methyldopa are the most prescribed antihypertensives in pregnancy [23]. Nifedipine is a calcium channel blocker which is effective at reducing blood pressure without uteroplacental blood flow nor slowing foetal heart rate [24]. While methyldopa is preferred because it is a centrally acting *α*-agonist that decreases sympathetic outflow to decrease BP, it has a very long duration of action and the best safety profile amongst the antihypertensive drugs used during pregnancy [25]. Currently methyldopa has no associated congenital anomaly. The prescribed medications are accepted for the management of hypertensive disorders in pregnancy because of their low risk of adverse complications in pregnancy. All the prescribed drugs are in line with the stated guidelines.

The mean blood pressure of pregnant women at 16 week gestation was 119.7/76.9 mmHg, which is considered normal. However, this average is confounded by several factors such as the gestational stage and the baseline blood pressure at the start of the pregnancy. At around the fifth week of gestation, there is an expansion of the blood volume which causes a drop in the blood pressure of the mother. At this critical stage, hypertensive mothers may appear to have a normal blood pressure [24]. This may have accounted for the normotensive mean blood pressure of mothers at that gestational age. At the point of delivery, it was realized that the mean BP of the mothers was 121.8/77 mmHg which saw a significant increase of the mean diastolic pressure of the mothers. Even though, the mean blood pressures of the mothers were still within the normotensive regions. This increase in the mean diastolic pressure may be normalization of the BP after the initial drop of the blood pressure.

Again, from the results obtained, it was realized that there was a significant decrease in the proportion of mothers who had high blood pressure by 10.4% (Table 1). This indicates that management of mothers who had high blood pressure at 16 week gestation resulted in a decrease of the proportion of mothers who had high blood pressure at the time of delivery. It is also worth noting that as part of management, some proportion of mothers delivered preterm as part of efforts to avert adverse birth complications. Thus, at time of delivery, these mothers would be hypertensive. Hence, although there was a drop, the proportion of mothers who were hypertensive at delivery was significantly higher and of public health concern.

The level of education influenced the risk of being hypertensive in pregnancy such that mothers who had junior high school education were 68% less likely to develop HDPs compared to the likelihood of mothers without any formal education developing HDPs. This finding is consistent with the findings of Abalos et al. [26] where evidence obtained established that education was a risk factor for hypertensive disorders. This may be that as an individual that is educated, there is an increased chance of been informed on the risk factors and prevention of HDPs. So that, they may take necessary measures to prevent the risk of developing HDPs. However, there was no statistical significance association between the odds of mothers who had no formal education developing HDPs compared to the odds of mothers who had other levels of education developing HDPs. Under the bivariate analysis, mothers with tertiary education were 1.85 times more likely to develop HDPs compared to mothers who had no formal education. However, after adjusting for known confounders, this association diminished with no statistical significance. This indicates that the observed association maybe an anomaly, and, as such, it is inconclusive to suggest that education status influences the risk of HDPs in this study.

It was also realized that high number of pregnancies greatly influenced the risk of HDPs (Table 4). Though several studies, such as Sengodan and Sreeprathi [27], have established that primigravid mothers were at risk of HDPs, the findings from this study corroborate the evidence by Cho et al. [28] that mothers with multiple gestation have an increased risk of hypertensive disorders in pregnancy, particularly preeclampsia. However, it has been established that up to about 61.2% of hypertensive disorders do not resolve after delivery [29]. When these hypertensions do not resolve before a woman gets pregnant again, it is considered as chronic hypertension in pregnancy and thus increases the cumulative burden of hypertensive disorders amongst mothers with multigravidity.

Genetics has been explained to influence the risk of chronic disease in several populations. The evidence of this study suggests that mothers who have family history of HDPs were at 3.6 times increased risk of developing HDPs compared to women who have no family history of hypertensive disorders. This is in sync with the systematic review by Tesfa et al. [30] which confirmed that family history of hypertension increased the risk of hypertensive disorders in pregnancy by fourfolds. Since family members share some genetic make-up and some lifestyle or habits [31], there is an increased likelihood that they may share similar risk of a particular disease burden [32]. This explains why mothers with family history of hypertensive disorders are at an increased risk of HDPs. It is therefore recommended that in diagnosing a mother of HDPs, the risk profile should consider the history of hypertension in the family. This would help in early diagnosing and management of hypertensive disorders.

The use of complementary and alternative medicine (CAM) is predominant in Ghana, and it is recognized by the health system [33]. Nonetheless, the role of CAM in management of HDPs is scarce [34]. Evidence from this study found that mothers who used herbal medicines were 2.9 times more likely to have HDPs. Some studies have reported the beneficial use of CAM in pregnancy and how it has improved on blood pressure levels and also on various obstetric outcomes [35].

This study's finding does not imply that the use of herbal medicines negatively increases blood pressure. This is because the study is methodologically limited to the use of herbal medicines. It did not assess the type of herbal medicine used, how the herbal medicines were used, and whether it was prescribed or was appropriate for use. However, this finding reveals a very critical issue of the appropriate use of herbal medicines in pregnancy. Herbal medicines are made up of several constituents, and the safety of their use should be ascertained. Currently, there is paucity of information on the use of herbal medicine in the management of hypertensive disorders, particularly in pregnancy. More research evidence would be needed to understand why pregnant women in selected health facilities use these medicines, its constituents, and appropriateness for use in hypertensive disorders.

Alcohol consumption is known to influence hypertensive disorders in adults. Several studies have propounded that alcohol consumption, particularly heavy consumption, increases the risk of hypertension in normotensive individuals [36]. From this study, alcohol consumers were 3.5 times more likely to experience HDPs compared to nonalcohol consumers. Alcohol has a biphasic effect on blood pressure. In less than 12 hours after consumption, alcohol reduces blood pressure, and subsequently, after 12 hours, alcohol tends to increase blood pressure. It is believed to influence the blood pressure through the renin-angiotensin-aldosterone system by increasing the concentration and activity of renin in blood [37]. An increase in renin is associated with an increase in a potent vasoconstrictor called angiotensin II, responsible for the rise in blood pressure. Additionally, alcohol is believed to reduce the baroreceptor sensitivity thus affecting blood pressure regulation at the long term [37]. It is advised that mothers must be counseled during ANC visits to strictly avoid alcohol intake during pregnancy.

The aetiology of these HDPs, however, is not known [38]. It is therefore recommended that to effectively reduce the incidence of HDPs, pregnant women should be counseled on the risk factors of these conditions and screened for during their antenatal care visits to prevent the adverse effects of its complications.

Some studies have established that HDPs influence the birth outcomes at delivery. From this study, mothers who had HDPs were 2.66 times more likely to have preterm babies. This finding is consistent with a study by Xiong et al. [39], where it was realized that HDPs increased the risk of stillbirth by twelvefolds. Hypertension is known as the leading cause of stillbirth by affecting the development of the placenta by restricting nutrient and oxygen flow to the foetus. Also, it increases the risk of abnormal bleeding between the placenta and uterine walls leading to placenta abruption [40]. All these causes foetal distress and subsequently death. Thus, it is critical to deliver the baby before term to avert foetal distress and possibly foetal wastage.

Another evidence from this study reveals that hypertensive mothers were 1.70 times more likely to undergo caesarean section. This finding is consistent with the findings of Roberts et al. [41], where it was realized that HDPs were associated with an increased risk of caesarean section amongst mothers during delivery. Considering this evidence, it is recommended that health professionals in charge of antenatal care critically monitor hypertensive mothers during care and to pragmatically help to control their blood pressure to normotensive status before delivery.

## 5. Conclusion and Recommendations

The overall prevalence of HDPs was 37.2% with chronic hypertension superimposed with preeclampsia having the highest prevalence of 17.6%. Lack of formal education, family history, multigravida, consumption of alcohol, and herbal preparation were identified as factors that could influence the risk of HDPs. The use oral nifedipine and methyldopa considerably reduced the proportion of mothers with high blood pressure. The selected mothers with HDPs were likely to undergo caesarean sections, experience stillbirth, or have preterm babies. The burden of HDPs is high amongst pregnant women seeking care in selected facilities. There is the need for intensified campaign on HDPs in the Ashanti Region of Ghana.

Considering the increasing risk of HDPs in the Ashanti region, its increased adverse association to obstetric outcomes such as stillbirth, preterm, and caesarean section, there is the need to investigate the cost impact of HDPs on the mother and the household. Additionally, the quality of life of mothers diagnosed with HDPs and the impact of HDPs on the risk of prenatal and postnatal depression should be assessed further.

## 6. Strength and Limitations

This study was well powered considering the sample size as such reducing the risk of type 1 error. Additionally, the participants were clearly defined using the inclusion and exclusion criteria, outcomes were appropriately measured, and potential confounding factors were considered in the analysis. Therefore, findings of this study are generalizable to pregnant women seeking antenatal care in the Sub-Saharan African region.

This study required mothers to recall past event, and this subjected the responses to recall bias. To minimize this bias, where mothers could not recall an event, they were permitted to skip the item on a questionnaire. Additionally, this study does not establish causality but rather provides the strength of association between the outcome variables and predictors, as such interpretations of the findings should be done carefully.

## Figures and Tables

**Table 1:** Sociodemographic characteristics of study respondents.

Variable	Without HDPs *n* (%)	With HDPs *n* (%)	Total *n* (%)
Age			
18–24	74 (23.57)	25 (13.44)	99 (19.80)
25–29	110 (35.03)	64 (34.41)	174 (34.8)
30–34	76 (24.20)	47 (25.27)	123 (24.6)
35–39	47 (14.97)	31 (16.67)	78 (15.6)
40–44	7 (2.23)	19 (10.22)	26 (5.20)
Educational background			
No education	51 (16.24)	29 (15.60)	80 (16.06)
Primary	23 (7.32)	7 (3.76)	30 (6.02)
JHS	81 (25.80)	26 (13.99)	107 (21.49)
SHS	83 (26.43)	46 (24.73)	129 (25.90)
Tertiary	76 (23.57)	78 (41.93)	154 (30.52)
Marital status			
Single	75 (23.89)	44 (23.66)	119 (23.90)
Married	236 (75.16)	140 (77.27)	376 (75.50)
Divorced	1 (0.32)	2 (1.08)	3 (0.60)
Religion			
Christian	246 (78.34)	163 (87.63)	394 (82.80)
Moslem	39 (12.42)	15 (8.06)	54 (10.93)
Others	24 (7.64)	7 (3.76)	31 (6.28)
Occupation			
Gov. employee	61 (19.43)	55 (29.57)	116 (23.08)
Housewife	38 (12.10)	22 (11.83)	60 (12.15)
Private business	209 (66.56)	109 (58.60)	320 (64.77)
Setting of facility receiving ANC			
Periurban	205 (65.28)	25 (13.44)	230 (46)
Urban	109 (34.71)	161 (86.56)	270 (54)
Knowledge			
Poor	63 (20.06)	6 (3.28)	69 (13.88)
Satisfactory	123 (39.17)	8 (4.37)	131 (26.36)
Good	40 (12.74)	38 (20.77)	78 (15.69)
Excellent	88 (28.03)	131 (71.58)	219 (44.06)

**Table 2:** Medical characteristics of study respondents.

Variable	Without HDPs *n* (%)	With HDPs *n* (%)	Total *n* (%)
Gravida			
Primigravida	96 (30.67)	37 (20.00)	133 (26.71)
Multigravida	204 (65.18)	125 (67.57)	329 (66.06)
Grand multigravida	13 (4.15)	23 (12.43)	36 (7.23)
Miscarriage			
No	260 (82.80)	140 (75.68)	400 (80.16)
Yes	54 (17.20)	45 (24.32)	99 (19.84)
Had caesarean section			
No	219 (69.75)	99 (53.23)	318 (63.60)
Yes	95 (30.25)	87 (46.77)	182 (36.40)
Anxious of pregnancy			
No	239 (76.11)	105 (56.76)	344 (68.94)
Yes	75 (23.96)	80 (43.24)	155 (31.06)
Alcohol consumption			
No	297 (95.19)	128 (69.19)	425 (85.51)
Yes	15 (4.81)	57 (30.81)	72 (14.49)
History of chronic condition			
No	279 (90.29)	144 (78.26)	423 (85.80)
Yes	30 (9.71)	40 (21.74)	70 (14.20)
Family history of HDPs			
No	236 (75.40)	101 (54.30)	337 (67.54)
Yes	77 (24.60)	85 (45.70)	162 (32.46)
First antenatal visit			
First trimester	194 (61.98)	148 (80.00)	342 (68.67)
Second trimester	106 (33.87)	31 (16.76)	137 (27.51)
Third trimester	13 (4.15)	6 (3.24)	19 (3.82)
Number of ANC visit			
1–3 times	112 (36.01)	149 (80.11)	261 (52.52)
4–6 times	95 (30.55)	25 (13.44)	120 (24.14)
Above 7 times	104 (33.44)	12 (6.45)	116 (23.34)

**Table 3:** Effect of antihypertensive therapy on BP control among women with HDPs.

Variable	At 16 weeks	At delivery	Difference (95% CI)
Mean systolic BP	137.60 mmHg ± 2.11	137.24 mmHg ± 1.89	0.36 mmHg (-5.19–5.91)
Mean diastolic BP	93.24 mmHg ± 1.81	89.02 mmHg ± 1.46	4.22mmhg (0.39–8.05)
Prop. with high BP	37.2% ± 0.022	26.8% ± 0.020	10.4% (4.7%-16.14%)

**Table 4:** Factors influencing risk of hypertensive disorders of pregnancy.

Variable	Category	Frequency (%)	COR	95% CI	AOR	95% CI
Age	18–24	99 (19.8)	Ref		Ref	
	25–29	147 (34.8)	1.72	1.00–2.98	1.82	0.82–4.06
	30–34	123 (24.6)	1.83	1.02–3.27	1.38	0.57–3.32
	35–39	78 (15.8)	1.95	1.03–3.71	1.21	0.45–3.23
	40-44	26 (5.2)	8.03	3.02–21.36	1.71	0.43–6.92

Residence	Periurban	230 (46.0)	Ref		Ref	
	Urban	270 (54.0)	7.53	2.27–24.91	1.95	0.46–8.34

Educational status	No education	80 (16.0)	Ref		Ref	
	Primary school	30 (6.0)	0.54	0.20–1.40	0.74	0.19–2.95
	Junior high school	107 (21.4)	0.56	0.30–1.06	**0.27**	**0.10–0.68**
	Senior high school	129 (25.8)	0.97	0.55–1.74	0.44	0.19–1.02
	Tertiary	154 (30.8)	1.85	1.06–3.23	0.53	0.20–1.43

Occupation	Gov. employee	116 (23.2)	Ref		Ref	
	Housewife	66 (12.0)	0.64	0.34–1.22	1.83	0.60–5.61
	Private Business	318 (63.6)	0.58	0.38–0.89	2.14	0.89–5.12

Had caesarean section	Yes	182 (36.4)	2.03	1.39–2.95	1.44	0.72–2.88

Anxious of pregnancy	Yes	155 (31.0)	2.13	1.64–3.59	1.24	0.61–2.55

History chronic condition	Yes	70 (14.0)	2.58	1.54–4.32	2.66	1.21–5.82

Gravida	Primigravida	133 (26.6)	Ref		Ref	
	Multigravida	329 (65.8)	1.59	1.02–2.47	1.34	0.70–2.53
	Grand multigravida	38 (7.6)	4.59	2.11–10.00	**4.53**	**1.42–14.42**

History of HDPs	Yes	160 (32.0)	2.58	1.54–4.32	**3.61**	**1.89–6.90 ** ^∗^

First antenatal visit	First trimester	342 (68.4)	Ref		Ref	
	Second trimester	137 (27.4)	0.38	0.24–0.60	0.60	0.21–1.72
	Third trimester	21 (4.2)	0.60	0.22–1.63	2.44	0.32–18.90

Number of antenatal care visit	1–3 times	261 (52.2)	Ref		Ref	
	4–6 times	120 (24.0)	0.20	0.12–0.33	0.81	0.35–1.89
	Above 7 times	119 (23.8)	0.09	0.05–0.17	**0.26**	**0.10–0.67**

Average monthly salary	Less than ȼ400	79 (15.8)	Ref		Ref	
	ȼ400–ȼ799	194 (38.8)	0.73	0.39–1.39	0.67	0.27–1.67
	ȼ800–ȼ1200	128 (25.6)	3.03	1.62–5.70	1.52	0.57–4.04
	More than ȼ1200	99 (19.8)	8.45	4.26–16.76	4.51	1.48–13.76

Herbal medication consumption	Yes	257 (51.4)	9.17	5.86–14.35	**2.92**	**1.15–7.41 ** ^∗^

Alcohol consumption	Yes	72 (14.4)	8.82	4.81–16.15	**3.53**	**1.42–8.78 ** ^∗^

Source of information	Health professionals	443 (88.6)	Ref		Ref	
	Family & friends	15 (15.0)	0.54	0.14–2.01	0.79	0.15–4.12
	Media	25 (5.0)	2.06	0.75–5.65	2.92	0.72–11.89
	Others	17 (3.4)	0.11	0.01–0.82	0.33	0.03–3.20

Monitoring of blood	No	20 (16.0)	Ref		Ref	

Pressure during ANC	Yes	436 (87.2)	5.75	1.32–25.10	2.19	0.24–19.81
	Not sure	44 (8.8)	3.30	0.66–16.61	10.38	0.92–117.11

Adjusted for all other variables shown. ^∗^Statistically significant. COR = crude odds ratio. CI = confidence interval. AOR = adjusted odds ratio. The entries in boldface are significant and must bear the symbol “^∗^” as shown in the table.

**Table 5:** Predictors of risk of preterm delivery.

Variable	Category	COR	95% CI	AOR	95% CI
Setting of ANC facility	Periurban	Ref		Ref	
	Urban	1.90	1.06–3.39	1.09	0.52–2.26

Hypertensive status	Not hypertensive	Ref		Ref	
	Hypertensive	3.15	1.79–5.55	**2.66**	**1.29–5.89 ** ^∗^

Alcohol consumption	Not consumer	Ref		Ref	
	Consumer	2.13	1.10–4.13	1.27	0.61–2.62

Family history of HDPs	No history	Ref		Ref	
	History	2.02	1.16–3.52	1.63	0.91–2.92

Adjusted for all other variables shown. ^∗^Statistically significant. COR = crude odds ratio. CI = confidence interval. AOR = adjusted odds ratio.

**Table 6:** Predictors of caesarean section.

Variable	Category	COR	95% CI	AOR	95% CI
Hypertensive status	Not hypertensive	ref		ref	
	Hypertensive	2.59	1.76–3.81	**1.70**	**1.10–2.61 ** ^∗^

Alcohol consumption	Not consumer	ref		ref	
	Consumer	5.49	3.19–9.45	**4.21**	**2.37–7.49 ** ^∗^

Family history of HDPs	No history	ref		ref	
	History	1.89	1.28–2.80	**1.61**	**1.06–2.46 ** ^∗^

Adjusted for all other variables shown. ^∗^Statistically significant. COR = crude odds ratio. CI = confidence interval. AOR = adjusted odds ratio.

**Table 7:** Predictors of stillbirth.

Variable	Category	COR	95% CI	AOR	95% CI
Age	18–24	Ref		Ref	
	25–29	1.15	0.28–4.70	0.82	0.19–3.58
	30–34	1.64	0.40–6.73	0.94	0.20–4.34
	35–39	1.73	0.38–7.97	1.04	0.21–5.16
	40–44	7.62	1.69–34.39	2.37	0.46–12.19

History of C/S	No	Ref		Ref	
	Yes	3.10	1.33–7.24	1.25	0.34–4.55

Anxious of pregnancy	No	Ref		Ref	
	Yes	3.31	1.43–7.62	1.04	0.26–4.17

History of chronic condition	No	Ref		Ref	
	Yes	2.87	1.14–7.26	1.72	0.62–4.77

Hypertensive status	Not hypertensive	Ref		Ref	
	Hypertensive	20.86	4.85–89.81	**12.47**	**2.72–57.24 ** ^∗^

Alcohol consumption	Not consumer	Ref		Ref	
	Consumer	5.70	2.44–13.30	2.02	0.58–7.12

Adjusted for all other variables shown. ^∗^Statistically significant. COR = crude odds ratio. CI = confidence interval. AOR = adjusted odds ratio.

## Data Availability

All the data obtained during this study are available from the corresponding author upon request.
